# Safety Outcomes After Sacubitril/Valsartan Initiation in a Community Teaching Hospital: A Retrospective Observational Study

**DOI:** 10.7759/cureus.65603

**Published:** 2024-07-28

**Authors:** Erin Anderson, Jessica Jones, Trager Hintze, Nephy Samuel

**Affiliations:** 1 Department of Pharmacy Practice and Translational Research, College of Pharmacy, University of Houston, Houston, USA; 2 Department of Pharmacy, St. Joseph Health Regional Hospital, Bryan, USA; 3 Department of Pharmacy Practice, Irma Lerma Rangel School of Pharmacy, Texas A&M University, College Station, USA

**Keywords:** blood glucose, glycemic changes, efficacy, safety, sacubitril/valsartan

## Abstract

Background: Sacubitril/valsartan is one of the main foundational medications in heart failure (HF), and recent data show that it provides a mortality benefit in this patient population. Some of the main safety concerns associated with this drug class include hypotension, hyperkalemia, angioedema, and acute kidney injury (AKI). Other studies have focused on sacubitril/valsartan effects on blood glucose levels, which is a newer concept that has not been as researched. This study seeks to explore the safety of angiotensin receptor-neprilysin inhibitors (ARNi) when initiated in acute hospitalized patients, where data on tolerability will assist in patient selection and monitoring. The results of this study will help distinguish the HF population at a community teaching hospital and aid clinician decision-making.

Methods: This was a single-center, retrospective, observational review utilizing electronic health records. To be included in this study, patients had to be 18 years or older with an HF diagnosis and must have taken at least two doses of sacubitril/valsartan before discharge. Primary safety outcomes will include discontinuation of sacubitril/valsartan, incidence of angioedema, hyperkalemia, AKI, hypotension, and hypoglycemia. Secondary outcomes included hospital length of stay and 30-day readmission rate.

Results: Data were collected for a total of 227 patients. AKI was the most common primary safety adverse effect captured with 30 patients (14.02%), followed by hypotension in 20 patients (9.35%), and hyperkalemia in 19 patients (8.9%). Only one patient (0.44%) experienced both angioedema and hypotension, and it happened to be the same patient. Additionally, only one patient (0.47%) was found to be hypoglycemic. Sacubitril/valsartan was discontinued in 41 patients (18.1%), and 20 of those patients (8.8%) were restarted at a lower dose. Lastly, 63 patients (27.8%) were readmitted within 30 days of discharge. Patents that also had diabetes had worse safety outcomes and were associated with 30-day readmission.

Conclusions: This study of patients admitted with HF and started on sacubitril/valsartan found similar rates of efficacy and safety outcomes to previously published reports. However, the rate of hypoglycemia associated with sacubitril/valsartan was low in only one patient, and it was strongly associated with previous antidiabetic medication use. This differs from previously published reports and analyses on the association of sacubitril/valsartan and hypoglycemia. More research is needed for the argument that sacubitril/valsartan has an effect on glycemic outcomes. Patients with diabetes were found to have worse safety outcomes and a higher 30-day readmission. Providers should consider monitoring these patients closely and making sure that patients are on guideline-directed medication therapy before discharge. Other common safety events associated with sacubitril/valsartan were also seen in this population.

## Introduction

Approximately 6.2 million adults in the United States have heart failure (HF), and it is estimated that the prevalence will increase by 46% from 2012 to 2030 [1]. Fortunately, with current research and guidelines, pharmacotherapy for HF patients has progressed. According to the 2022 AHA Heart Failure Guidelines, angiotensin receptor-neprilysin inhibitors (ARNi) are recommended as first-line therapy over angiotensin-converting enzyme inhibitors (ACEi) or angiotensin 2 receptor blockers (ARB) in HF with reduced ejection fraction (EF) (HFrEF) patients [2]. ARNi benefit HF with midrange EF (HFmrEF) and HF with preserved EF (HFpEF) patients, so they are recommended in these HF subsets as well. The trial performed by McMurray et al. was a major proponent for the addition of ARNi agents to the guidelines, showing a reduction in cardiovascular death or HF hospitalization with sacubitril-valsartan vs. enalapril in symptomatic patients with HFrEF [3].

Sacubitril/valsartan use has increased in our HF population, due to this addition to guideline-directed therapy. However, like all medications, it does have adverse effects such as hypotension, hyperkalemia, angioedema, and acute kidney injury (AKI), and according to some recent literature, it may also have some effect on blood glucose levels [4]. Presently, there is not much known regarding the safety and tolerability of sacubitril/valsartan when started in the hospital. There are studies that shed light on the safety and tolerability of ARNi use, but they are not without limitations. Some studies, like the one performed by McMurray et al., required a four- to six-week run-in period, which excluded treatment-naive patients [3]. Other studies had a short in-hospital monitoring period after initiation, which makes capturing some safety outcomes difficult [5]. Others have only focused on specific side effects and did not capture others such as hypoglycemia with sacubitril/valsartan, which has been less studied [5-9]. Lastly, some had small sample sizes and/or excluded HFmrEF or HFpEF patients [3-5,7]. This study was designed to add to current research by targeting some of those limitations, capturing proposed glycemic outcomes, and characterizing patients at risk for poor safety outcomes.

## Materials and methods

This was a single-center, retrospective, observational review utilizing electronic health records. The study was deemed exempt by the authors' local institutional review board (#1977729-2). To be included in this study, patients had to be 18 years or older with an HF diagnosis (per diagnosis code) and must have taken at least two doses of sacubitril/valsartan before discharge. Exclusion criteria included pregnant or nursing patients, prisoners, and previous use of sacubitril/valsartan. Theradoc© was used to screen patients at St. Joseph Regional Health Hospital between January 1, 2019, and August 10, 2022, that had sacubitril/valsartan administrations, which resulted in 950 patients. These dates were chosen as the medication was added to the hospital formulary on January 1, 2019. A total of 325 duplicate patients were removed for a total of 625 patients, 112 of which only had one administration, so they were also removed, leaving 513 patients. Patients were then filtered down to new start sacubitril/valsartan, by checking if the medication was on their home medication list on admission and removing previous users. The final patient list included 227 patients in total.

The primary objective of this study is to assess the safety and tolerability of sacubitril/valsartan when initiated in HF patients. Primary safety outcomes will include discontinuation of sacubitril/valsartan, the incidence of angioedema, and changes in potassium (K), serum creatinine (SCr), systolic blood pressure (SBP), and blood glucose (BG) from baseline. The incidence of hyperkalemia was tracked and defined as serum potassium greater than five after starting sacubitril/valsartan. The incidence of AKI was also assessed and defined as a rise in SCr of 0.3 or more. Lastly, the incidence of hypotension was tracked and defined as an SBP less than 90 mmHg, and hypoglycemia was defined as BG less than 60 mg/dL. Secondary outcomes included hospital length of stay and 30-day readmission rate.

Data collection also included other HF medications already on patient profiles on admission, to better describe the patient population. HF medications screened for included the following: ACEi, ARB, sodium-glucose cotransporter 2 inhibitors (SGLT2i), digoxin, beta-blockers, isosorbide dinitrate and hydralazine HCL, ivabradine, vericiguat, loop diuretics, and mineralocorticoid receptor antagonists (MRAs). A history of insulin or other oral antidiabetic medications was noted to detect causes of hypoglycemia other than sacubitril/valsartan. Continuation of any and/or all home medications was at the discretion of the individual provider. The presence of a washout period if previously on an ACEi was assessed, as well as the use of vasopressors or IV diuretic use. Characteristics collected also included the ejection EF, age, race and ethnicity, body mass index (BMI), gender, comorbidities, date of admission and discharge, time to sacubitril/valsartan initiation, dose and frequency of sacubitril/valsartan, administrations of sacubitril/valsartan during admission, angioedema incidence, and periodic levels of serum K, SCr, BG, and SBP. Many of these characteristics allowed for exploratory subgroup analysis after data collection.

Descriptive statistics were used to describe the baseline characteristics, rates of the primary safety outcomes, and secondary outcomes of discontinuation and 30-day readmission. The 30-day HF readmission rate was compared to the national average of about 25% [10,11]. Subgroup statistical analysis was conducted using Pearson chi-square tests for categorical variables, Fisher’s exact test was utilized for outcomes with small sample sizes, and two sample t-tests were conducted for continuous variables. Excel (Microsoft® Corp., Redmond, WA) data software was used. All studies were conducted using STATA statistical software (version 17; StataCorp LLC., College Station, TX). All statistical tests were two-sided, and statistical significance was determined at a p-value < 0.05.

## Results

Baseline characteristics and patient demographics are included in Tables 1-2. Data were collected for a total of 227 patients. The mean age of the patients was 70 years; 61.7% were male, and 67.4% were White. The most common comorbidities were diabetes, hypertension, and coronary artery disease (CAD), with an average EF of 28.8%. The majority of patients (93.8%) started on sacubitril/valsartan 24-26 mg twice daily, and a smaller portion (6.2%) started on the 49-51 mg twice daily dose. Only one patient did not have a proper washout period of 36 hours between the ACEi discontinuation and the first dose of sacubitril/valsartan. The most common heart failure home medications were beta-blockers, ACEi, and loop diuretics. Most diabetic patients were on insulin glargine or a sulfonylurea at home. While in the hospital, 68.7% of patients received intravenous (IV) loop diuretics, whereas only 12.3% were on vasopressin or norepinephrine.

**Table 1:** Baseline characteristics ^1^Body mass index; ^2^Estimated glomerular filtration rate

Variable	New-Start Sacubitril/Valsartan Patients (n = 227) Average + Range
Mean age + range (yr.)	66.95 (25-97)
BMI^1^ (kg/m^2^)	30.41 (17.5-59.3)
Days of therapy	3.8 (1-16)
eGFR^2^ (mL/min/1.73 m^2^)	65.1 (0-95)
Ejection fraction (EF)	28.8% (5-70%)

**Table 2:** Patient demographics *The time between the last dose of ACEi and ARNI was 11 hours. ^1^Mineralocorticoid receptor antagonist; ^2^Angiotensin converting enzyme inhibitors; ^3^Angiotensin 2 receptor blockers; ^4^Sodium-glucose cotransporter 2 inhibitors

Variable	n (%)
Male sex	140 (61.7%)
Caucasian/White	153 (67.4%)
African American	48 (21.1%)
Hispanic or Latino	22 (9.7%)
Asian	1 (0.44%)
American Indian/Alaska Native	1 (0.44%)
Other	2 (0.88%)
History of diabetes	82 (36.1%)
History of cirrhosis	2 (0.88%)
History of hypertension	166 (73.1%)
History of chronic kidney disease	29 (12.8%)
History of previous myocardial infarction	13 (5.7%)
History of pacemaker	43 (18.9%)
History of atrial fibrillation	50 (22%)
History of stroke	12 (5.3%)
History of coronary artery disease	66 (29.1%)
Initiation dose/frequency of sacubitril/valsartan 24-26 mg twice daily	213 (93.8%)
Initiation dose/frequency of sacubitril/valsartan 49-51 mg twice daily	14 (6.2%)
Absence of washout period	1* (0.44%)
Intravenous loop diuretic on medication profile during visit	156 (68.7%)
Received vasopressors for hypotension during hospitalization	28 (12.3%)
Loop diuretic on home medication list	79 (35.9%)
Beta-blocker on the home medication list	109 (48%)
Digoxin on the home medication list	7 (3.1%)
MRA^1^ on the home medication list	24 (10.9%)
ACEi^2^ on the home medication list	51 (23.1%)
ARB^3^ on the home medication list	24 (10.9%)
SGLT2i^4^ on the home medication list	5 (2.3%)
Insulin on the home medication list	34 (14.98%)
Sulfonylurea on the home medication list	23 (10.1%)
Thiazolidinedione on the home medication list	2 (0.88%)

Primary and secondary outcomes are included in Table 3. AKI was the most common primary safety adverse effect captured in 30 patients (14.02%), followed by hypotension in 20 patients (9.35%) and hyperkalemia in 19 patients (8.9%). Only one patient (0.44%) experienced both angioedema and hypotension, and it happened to be the same patient. Sacubitril/valsartan was discontinued in 41 patients (18.1%), and 20 of those patients (8.8%) were restarted at a lower dose. Lastly, 63 patients (27.8%) were readmitted within 30 days of discharge. For the hypotension and hypoglycemia outcomes, the study was designed to try to control for various confounding factors that could contribute to the outcome. Hypotensive patients were checked for the presence of an IV loop diuretic on the medication administration record during the visit, as well as vasopressor use. Fifteen of the 20 patients (75%) who experienced hypotension were also on IV loop diuretics (P = 0.494), and two of the 28 (7.1%) patients on vasopressin/NE experienced hypotension (P = 0.884). All of the patients who did experience hypotension were started on the lower 24-26 mg BID dose of sacubitril/valsartan. The patient on insulin detemir experienced hypoglycemia and was not on any other antidiabetic medications such as thiazolidinediones or sulfonylureas. However, it is unclear what antidiabetic medications the patient was on while in the hospital, and if they were titrated while inpatient. The one patient with an improper washout period discontinued sacubitril/valsartan afterward, but did not experience any of the monitored safety adverse events.

**Table 3:** Primary and secondary outcomes

Outcomes	n (%)
Angioedema	1 (0.44%)
Hyperkalemia	19 (8.9%)
Acute kidney injury	30 (14.02%)
Hypotension	20 (9.35%)
Hypoglycemia	1 (0.47%)
Discontinuation (secondary outcome)	41 (18.1%)
Restarted at a lower dose (secondary outcome)	20 (8.8%)
30-day readmission rate (secondary outcome)	63 (27.8%)

Exploratory subgroup analysis results are summarized in Table 4. For patients who experienced hypotension, there was a statistically significant relationship between a past medical history of atrial fibrillation (Afib) (P = 0.015) and having insulin on the patient’s home medications (P = 0.045). The one patient who was hypotensive/had angioedema was on insulin detemir and digoxin at home (P < 0.001) and had a pacemaker (P = 0.048). The one patient with angioedema also experienced hypoglycemia, which is why many subgroups are correlated, and the patient did have a proper washout period. AKI was associated with insulin glargine (P = 0.047), detemir (P = 0.036), glyburide (P = 0.036), SGLT2i (P = 0.013), and hydralazine (P = 0.013) on patient’s home medications. Hyperkalemia and mean difference in eGFR were also statistically significant when related to AKI (P = 0.003; P = 0.0013). Hyperkalemic patients were associated with insulin glargine (P = 0.039), glyburide (P = 0.004), pioglitazone (P = 0.044), and Bidil (P = 0.044) on their home medications, as well as sacubitril/valsartan on their active medications (P = 0.040). Lastly, the patients readmitted within 30 days of discharge were associated with a past medical history of diabetes (P = 0.022), mean difference in age (P = 0.0019), and mineralocorticoid receptor antagonists (MRA) on their home medication list (P < 0.001).

**Table 4:** Exploratory subgroup analysis Bold text = statistically significant P value ^1^Past medical history; ^2^Acute kidney injury; ^3^Sodium-glucose cotransporter 2 inhibitors; ^4^Estimated glomerular filtration rate; ^5^Mineralocorticoid receptor antagonist; ^6^Angiotensin 2 receptor blocker

Outcome n (%)	Subgroups Associated	P value
Hypotension 20 (9.35%)	PMH^1^ of diabetes	P = 0.093
	PMH^1^ atrial fibrillation	P = 0.015
	Insulin on the home medication list	P = 0.045
	Digoxin on the home medication list	P = 0.069
	Hyperkalemia	P = 0.066
Hypoglycemia 1 (0.47%)	PMH^1^ of hypertension	P = 0.08
	PMH^1^ of pacemaker	P = 0.048
	PMH^1^ of atrial fibrillation	P= 0.068
	Insulin detemir on the home medication list	P < 0.001
	Digoxin on the home medication list	P < 0.001
	Angioedema	P < 0.001
AKI^2^ 30 (14.02%)	Insulin glargine on the home medication list	P = 0.047
	Insulin detemir on the home medication list	P = 0.036
	Glyburide on the home medication list	P = 0.036
	SGLT2i^3^ on the home medication list	P = 0.013
	Hydralazine on the home medication list	P = 0.013
	Hyperkalemia	P = 0.003
	Mean difference in eGFR^4^	P < 0.001
Hyperkalemia 19 (8.9%)	Insulin glargine on home medication list	P = 0.039
			Glyburide on the home medication list	P = 0.004
	Glipizide on the home medication list	P = 0.072		
			Pioglitazone on the home medication list	P = 0.044
	Isosorbide dinitrate/ hydralazine on home meds	P = 0.044		
			Hypotension	P = 0.066
	Sacubitril valsartan 24-26 mg dose	P = 0.088		
			Sacubitril valsartan 49-51 mg dose	P = 0.040
	Mean difference in eGFR^4^	P = 0.0886		
			Angioedema 1 (0.44%)	PMH^1^ of pacemaker	P = 0.040
	PMH^1^ of atrial fibrillation	P = 0.061	
	Insulin detemir on the home medication list	P < 0.001	
	Digoxin on home medication list	P < 0.001	
	30-day readmission rate 63 (27.8%)	Sacubitril/Valsartan 24-26 mg on the home medication list	P = 0.059
		Vasopressor administered during visit	P = 0.070
		PMH^1^ Diabetes	P = 0.022
		MRA^5^ on the home medication list	P < 0.001
		ARB^6 ^on the home medication list	P = 0.204
Mean difference of age		P = 0.0019
Mean difference in the duration of treatment		P = 0.0927

## Discussion

In this single-center, retrospective, observational review of patients hospitalized and started on sacubitril/valsartan, common side effects were observed, but there was no association between glycemic changes and therapy initiation. These findings contradict pooled analysis trials that included PARADIGM-HF (Prospective Comparison of Angiotensin Receptor Neprilysin Inhibitor With an Angiotensin-Converting Enzyme Inhibitor to Determine Impact on Global Mortality and Morbidity in Heart Failure) and PARAGON-HF (Prospective Comparison of Angiotensin-Converting Enzyme Inhibitor With Angiotensin Receptors Blockers Global Outcomes in Heart Failure With Preserved Ejection Fraction) patients [8,9] that compared changes in hemoglobin A1C, and new initiation of oral antihyperglycemic drugs or insulin in patients started on sacubitril/valsartan compared to either enalapril or valsartan. The most common safety events seen were hypotension, hyperkalemia, and AKI. Patients on concurrent diabetes medications had poorer safety outcomes and a higher 30-day readmission rate. The readmission rate at this small community hospital of 27.8% was slightly larger than the average 30-day readmission rate for HF patients, which can be up to 25% [10,11].

The purpose of this study was to add to current research by targeting previous study limitations, capturing proposed glycemic outcomes, and conducting post-hoc/exploratory sub-group analysis to better understand patients at risk for poor outcomes. Compared to the studies conducted by McMurray et al. and Velasquez et al. [3,6], the rates of adverse effects (hypotension, hyperkalemia, and AKI) seen in this study were similar, even though there was a smaller patient population and slightly varied definitions of the outcomes collected. As for the glycemic outcomes, Wijkman et al. conducted a pooled analysis of the McMurray et al. and Velasquez et al. studies compared to hypoglycemic events in their study [3,4,6]. The comparison was difficult given that all of the studies used slightly different measures for hypoglycemia with some employing the use of hemoglobin A1c and some using blood glucose monitoring. The main takeaway from comparing glycemic outcomes in these two studies is that this safety outcome needs to be studied further. Uniform outcome measurements for hypoglycemia, changes in HbA1c, and initiation of new diabetic medications would also improve clinical applicability. Some limitations of the pooled analyses study include investigator-reported hypoglycemia without biochemical confirmation, only including diabetic patients, no analysis of dose adjustments or changes in antihyperglycemic medications, and changes in hemoglobin A1c were observed against the background of therapeutic treatment decisions that took place outside of the study protocol. Another consideration is whether to measure glycemic outcomes in diabetic patients only, vs. all HF patients. Keeping these two populations separate for comparison could also be useful. In the Wijkman et al. and McMurray et al. studies that analyzed all patients (with or without diabetes) who did not use glucose-lowering medications at baseline, there was no significantly increased risk for hypoglycemia observed in the sacubitril/valsartan group (0.2% vs. 0.1%; P = 0.24) [3,4]. This is important because the current study included both diabetic and non-diabetic patients. If only diabetic patients (82 patients in this population of 227) were included in a subgroup analysis, the incidence of hypoglycemia would be 1.2% vs. 0.47%.

In the exploratory subgroup analysis, patients on concurrent diabetic medications had a higher incidence of all five safety outcomes, as well as the 30-day readmission rate. Diabetes is a common comorbidity for HF patients, which is associated with reduced survival and increased risk for HF hospitalization or cardiovascular death [8]. Poor glycemic control has been associated with an increased risk of HF hospitalization [8], and the requirement for insulin has been associated with an increased risk for adverse cardiovascular outcomes in HF patients. Macrovascular diabetic complications include diabetic nephropathy, which could also explain the association between hyperkalemia and AKI safety outcomes [12-15]. Therefore, it is not surprising that patients with additional comorbid conditions, especially diabetes, would experience worse outcomes. Keeping these risks in mind, providers should consider monitoring these patients more closely. This could include labs (particularly those for blood glucose monitoring) being ordered more often, starting at the lower dose of sacubitril/valsartan, and more frequent vitals. 

The association between HF medications such as MRA, ARB, and ARNi on home medications and the 30-day readmission rate makes sense considering that it is recommended to start patients on guideline-directed medication management before discharge for HF exacerbations [2]. Of note, patients with MRA on their home medication list did not have any readmissions during the 30-day time period. According to the ACC/AHA 2022 Heart Failure Guidelines, MRA have shown to improve all-cause mortality, HF hospitalizations, and sudden cardiac death in HFrEF patients [2]. It currently stands as one of the guideline-directed medical therapy agents in HFrEF and also has a 2b recommendation in the HFmrEF and HFpEF patient populations [2]. This study adds to the agreement that MRA decrease HF hospitalizations, as indicated by the 30-day readmission rate in this subgroup, and emphasizes the importance of initiating patients on this medication before discharge if they meet the criteria. This is in contrast to patients who were on vasopressin or norepinephrine during their stay, who were associated with a higher 30-day readmission rate. Patients who experience hemodynamic instability requiring vasopressors generally are worse clinically than those who do not, so the higher admission rate in this population was not surprising.

This study is not without limitations. The retrospective study design limits the study in that it was limited to information contained within the EMR and relied heavily on variable documentation; further, it does not allow for control of potential confounding factors nor allows for more long-term follow-up. The small sample size of 227 patients, while strong for a single-center study, does present an inherent risk of type II error. Originally, safety outcomes were going to be measured as an overall change from baseline during different times of hospitalization. This was not captured since the hospital records, which often lack all the clinical details necessary to appropriately classify, had missing or inconsistent data. Therefore, safety outcomes were reported as meeting certain cutoffs instead (hyperkalemia, hypotension, etc.). Other glycemic outcomes, uniform blood glucose levels, and distinction between diabetic and non-diabetic patients could have improved the generalizability of the hypoglycemia outcome. Non-uniform blood glucose values (fasting vs. non-fasting) were another limitation of this study, but due to the retrospective nature of the study and lack of a specific nursing requirement, this was difficult to control for. This study also did not collect information on new start insulin, just whether or not patients were on diabetes medications at baseline. We did our best to control for confounders such as antihyperglycemic agents on patient home medication lists, but tracking the addition and dose changes of new insulin or antihyperglycemic agents was out of the scope of this study. Lastly, this study lacked other glycemic outcomes such as hemoglobin A1C. Considering the short amount of time patients were monitored in the hospital, measuring this outcome would have been inappropriate since it measures changes in glycemia over a three-month period. Given the information from this study, future prospective studies are warranted, particularly those with larger sample sizes and longer follow-up periods. This would allow for a more comprehensive assessment of the association between sacubitril/valsartan and glycemic control.

## Conclusions

This study of patients admitted with HF and started on sacubitril/valsartan found that there was no association between sacubitril/valsartan and hypoglycemia. More research is needed for the argument that sacubitril/valsartan has an effect on glycemic outcomes. Patients with diabetes were found to have worse safety outcomes and a higher 30-day readmission. Providers should consider monitoring these patients closely and making sure that patients are on guideline-directed medication therapy. Other common safety events associated with sacubitril/valsartan were also seen in this population.
